# Methionine enkephalin (MENK) protected macrophages from ferroptosis by downregulating HMOX1 and ferritin

**DOI:** 10.1186/s12953-024-00228-x

**Published:** 2024-01-20

**Authors:** Jing Tian, Wenrui Fu, Zifeng Xie, Yuanlong Zhao, Haochen Yang, Jiafan Zhao

**Affiliations:** 1 Department of Immunology, School of Basic Medical Science, Jinzhou Medical University, Jinzhou, Liaoning 121000 China; 2 Graduate College, Jinzhou Medical University, Jinzhou, Liaoning 121000 China; 3 First Clinical Medical College, Jinzhou Medical University, Jinzhou, Liaoning 121000 China

**Keywords:** MENK, Macrophage, Proteomics analysis, Ferroptosis, Iron metabolism

## Abstract

**Objective:**

The aim of this work was to investigate the immunological effect of MENK by analyzing the protein spectrum and bioinformatics of macrophage RAW264.7, and to explore the relationship between macrophage and ferroptosis.

**Result:**

We employed proteomic analysis to identify differentially expressed proteins (DEPs) between macrophages and macrophages intervened by MENK. A total of 208 DEPs were identified. Among these, 96 proteins had upregulated expression and 112 proteins had downregulated expression. Proteomic analysis revealed a significant enrichment of DEPs associated with iron metabolism. The identification of hub genes was conducted using KEGG pathway diagrams and protein-protein interaction (PPI) analysis. The hub genes identified in this study include HMOX1 and Ferritin (FTH and FTL). A correlation was established between HMOX1, FTH, and FTL in the GO and KEGG databases. The results of PCR, WB and immunofluorescence showed that MENK downregulated the level of HMOX1 and FTH.

**Conclusion:**

MENK had the potential to become an adjuvant chemotherapy drug by regulating iron metabolism in macrophages, reducing levels of HMOX1 and ferritin. We proposed an innovative research direction on the therapeutic potential of MENK, focusing on the relationship between ferroptosis and macrophage activity.

**Supplementary Information:**

The online version contains supplementary material available at 10.1186/s12953-024-00228-x.

## Introduction

Cancer is widely recognized as one of the primary causes of mortality worldwide. According to a report released by the World Health Organization (WHO) on February 3, 2022, the number of deaths attributed to cancer in 2020 was approximately 10 million, accounting for nearly one in six deaths (https://www.who.int/news-room/fact-sheets/detail/cancer). Although cancer is primarily caused by the accumulation of genetic mutations, it should be noted that only 5–10% of cancer cases can be attributed to genetic defects, while the remaining 90–95% of cancer cases are believed to be influenced by environmental and lifestyle factors [1, 2].

In the initial stages of research, the focus of most researchers was primarily on tumor cells. Since Rosenberg found that platinum drugs had anticancer properties, the use of chemotherapeutic protocols has gained popularity [3, 4]. However, the persistent challenges faced by the medical community for several decades included the occurrence of severe adverse reactions and the development of drug resistance to platinum-based medications. With the advancement in cancer research, it has been acknowledged that cancer was a complex and dynamic process, characterized by a gradual evolution. This evolution was influenced by the reciprocal interactions between cancer cells and the microenvironment in which they reside [5].

The tumor microenvironment (TME) encompasses the entirety of the cellular milieu, comprising both the tumor cells and the immune cells in the surrounding vicinity [6, 7]. As certain drugs had the potential to impact the TME, experts proposed a novel approach to chemotherapy. This approach involved the use of immune-regulated drugs to activate immune cells against tumor development, thereby achieving the desired effect of immunotherapy. For instance, cancer vaccines have been regarded as a form of immunotherapeutic drug that activates immune cells rather than directly targeting tumor cells [8–10]. Some researchers summarized that vaccines stimulated the activation of innate immune cells, such as antigen presenting cells (APCs), which included dendritic cells (DCs) and macrophages, as well as adaptive immune cells like T cells [10–12]. The status of immune regulatory drugs witnessed significant improvement, garnering increased attention in the field of immunotherapy due to their ability to regulate the microenvironment. Over the course of time, researchers have made significant advancements in the field of TME, particularly in understanding the cellular metabolism of cancer cells as well as the immune cells in their vicinity.

Since the initial discovery of iron metabolism and its association with ferroptosis, as well as its potential anticancer properties, ion metabolism has emerged as a prominent area of interest among researchers studying tumors [13]. Gradually, researchers observed the process of iron metabolism and ferroptosis in the TME, and these findings were expected to greatly advance the field of cancer treatment. In recent studies, researchers have found that ferroptosis interacted with the immune system to some extent. This was because metabolism of tumor cells was vulnerable and there were variations in sensitivity among different cell states [14]. Immunotherapy encompassed various approaches, such as tumor immune checkpoint inhibitor therapy, activation of tumor immune cells, targeting tumor metabolism addiction, and induction of innate transformation of anticancer macrophages [14]. In the investigation of ferroptosis within the context of the TME, recent research has indicated that ferroptosis can trigger an inflammatory response. This response acted as an immunogenic trigger and played a role in regulating various forms of necrosis [15]. By investigating the regulation of immune cells and molecules, studying the process of ferroptosis in the tumor microenvironment (TME) would be valuable in the development of new immune-regulating drugs, offering a unique option for immunotherapy.

Methionine enkephalin (MENK) is an endogenous opioid that was first identified by Hughes in 1975, composed of Tyr-Gly-Gly-Phe-Met [16]. As a member of the endogenous opiate family, it has been regarded as a potent and long-lasting analgesic because of its ability to modulate pain sensitivity [17]. After the identification of opioid receptors on the membranes of immune cells, the modulatory effects of MENK were subsequently discovered [18]. Several articles reported that MENK possessed anticancer properties through its interaction with opioid receptors on immune cells and cancer cells [19, 20]. Our team has previously found that MENK has the ability to activate macrophages, thereby playing a significant role in both antiviral and anticancer mechanisms [21, 22]. All the findings suggested that MENK had a positive impact on immune inflammatory function, and importantly, it also modulated the tumor immune microenvironment. Furthermore, the study revealed that MENK demonstrated promising characteristics as an immune modulatory drug in the field of cancer immunotherapy. This made MENK a novel option for selecting chemotherapy regimens.

The role of macrophages as a link between the innate and adaptive immune responses has been extensively discussed in the context of the tumor immune microenvironment, covering aspects of pathogenesis and therapy. Tumor-associated macrophages (TAMs) are a specific type of macrophage that reside in the unique microenvironment of tumors. These TAMs can be modulated to adopt different polarization states, specifically M1 and M2, depending on the stimuli present in the local microenvironment [23]. This polarization influences their biological functions within the tumor. For both polarization states of M1 and M2 phenotypes, the M1 macrophage phenotype is associated with tumor regression, inhibition of tumor growth, and prevention of tumor occurrence. M2 macrophages hinder the anticancer functions of T cells and M1 macrophages by secreting pro-angiogenic substances and immunosuppressive cytokines [23, 24]. TAMs have been identified as a subtype of M2 phenotype due to their role in promoting tumor development, progression, and metastasis. This is achieved through the production of immunosuppressive cytokines, such as IL-10 and VEGF [25, 26]. Activating macrophages and inducing their transformation into an anticancer phenotype has been recognized as a promising avenue for immunotherapy. Given the recent findings in various researches, the correlation has been established between ferroptosis and macrophages. A potential avenue for investigating the pharmacological mechanism of immune-regulating drugs in immunotherapy is to explore the modulation of macrophages through ferroptosis. The main cause of Ferroptosis has been reported to be the reactive oxygen species (ROS) generated from the Fenton reaction and/or the catalysis of lipoxidase [27]. It also demonstrated that macrophages were capable of ROS, which can affect the condition of both tissue cells and the macrophages themselves by activating iron and lipid metabolism pathways. Iron accumulation played a role in promoting the M1 polarization of macrophages, which leaded to the release of inflammatory factors and the initiation of inflammation. Furthermore, certain reports have indicated that ROS play a role in polarizing macrophages towards the M1 phenotype, creating the conducive environment for the occurrence of ferroptosis [28, 29]. These findings indicate a close relationship between macrophages and ferroptosis.

MENK, as an immune modulatory drug, has shown significant improvement in the functionality of macrophages for both anticancer and antiviral purposes. At that particular juncture, our previous research demonstrated that MENK had the ability to activate macrophages, promoted M1 polarization, thereby enhanced immune function [30]. To investigate the molecular mechanisms in the immune microenvironment, the present study utilized proteomics analysis and bioinformatics analysis to identify differentially expressed proteins (DEPs) between macrophages and macrophages treated with MENK.

## Methods and materials

### Materials and reagents

Murine macrophage cell line (RAW264.7) was purchased from the Cell Resource of the Chinese Academy of Sciences (Shanghai, China). MENK (≥ 99% purity) was provided by American Peptide. Inc. One Step SYBR® Prime Script™ RT-PCR Kit was purchased from TaKaRa. The mAbs of HMOX1(ab189491) and FTH(ab75973) were purchased from Abcam. GAPDH(AF7021) was purchased from Affinity. FITC IgG (H + L)(RS0004) and HRP IgG (H + L)(RS0002) were purchased from Immunoway.

### Cell grouping

RAW264.7 cells were divided into two groups: Group A (the control group) and Group B (the MENK group). The cells in the control group were cultured in DMEM (Gibco, USA) supplemented with 10% fetal bovine serum (FBS; Gibco, USA), 100 U/mL penicillin, and 100 μg/mL streptomycin. The MENK group was treated with an optimal concentration of MENK (10 mg/mL) in DMEM medium. After 72 h, the cells were washed with PBS, and the cell pellets were obtained for proteomics analysis.

### Protein extraction and digestion

Added four times the volume of lysis buffer containing urea (8 M) and a 1% protease inhibitor cocktail to the samples. Sonication lysed the cells. The remaining debris was removed by centrifugation at 12,000 g for 10 min at 4 °C. The supernatant was collected and the protein concentration was determined using a bicinchoninic acid kit.

The protein solution was treated with dithiothreitol (5 mM) for 30 min at 56 °C, and then alkylated with iodoacetamide (11 mM) in the dark for 15 min at room temperature. Then, the protein sample was diluted with tetraethylammonium bromide (100 mM). Trypsin was added twice. The mass ratio of trypsin to protein was 1:50 for overnight digestion in the first round, and 1:100 for 4 h digestion in the second round.

### Liquid chromatography-tandem mass spectrometry (LC–MS/MS)

Tryptic peptides were dissolved in solvent A (0.1% formic acid, 2% acetonitrile in water) and loaded directly onto a homemade reversed-phase analytical column (length = 25 cm, 100 μm i.d.). Peptides were separated using a gradient from 4 to 23% solvent B (0.1% formic acid in 90% acetonitrile) over a period of 62 min. This was followed by a gradient ranging from 23 to 35% over a period of 20 min. It then increased to 80% within 4 min and remained at 80% for another 4 min. The entire process was carried out at a constant flow rate of 500 nL/min on an EASY-nLC 1200 UPLC system (Thermo Fisher Scientific, Waltham, MA, USA).

Separated peptides were analyzed on a Q Exactive™ HF-X mass spectrometer (Thermo Fisher Scientific) equipped with a nano-electrospray ion source. The electrospray voltage applied was 2.1 kV. The full MS scan resolution was set to 120,000 for a scan range of 400–1500 m/z. Selected the 10 most abundant precursors for further MS/MS analyses with a dynamic exclusion of 30 s. Higher-energy C-trap dissociation was performed with a normalized collision energy of 28%. Fragments were detected at a resolution of 15,000 in the Orbitrap. Set the automatic gain control target to 5E4, the intensity threshold to 2.5E5, and the maximum injection time to 40 ms.

### Database search

MaxQuant 1.6.15.0 (www.maxquant.org) was used to analyze the resulting MS/MS data. We searched tandem mass spectra against the Mus_musculus_10090_SP_20201214 database. The FASTA database concatenated with the reverse decoy database. Trypsin/P was specified as the cleavage enzyme, allowing a maximum of two missing cleavages. The mass tolerance for precursor ions was set at 20 ppm in the first search and 5 ppm in the main search. The mass tolerance for fragment ions was set to 0.02 Da. Carbamidomethyl on Cys was specified as a fixed modification. Acetylation on the protein N-terminus, oxidation on methionine, and deamidation (NQ) were specified as variable modifications. The false discovery rate was adjusted to be less than 1%. The minimum score for peptides was greater than 40.

### Protein-protein interaction (PPI) network

The protein-protein interactions were obtained from the STRING database with a confidence score of 0.7, and the network was visualized using Cytoscape 3.7.2.

### Bioinformatics analysis

Set thresholds for differentially expressed proteins (DEPs): “fold change > 1.50 or < 0.67 and *P* < 0.05”. DEPs were annotated separately into biological process (BP), cellular component (CC), and molecular function (MF) using the Gene Ontology (GO) database (http://geneontology.org/). Analyzed the enrichment of signaling pathways using the Kyoto Encyclopedia of Genes and Genomes (KEGG) database (www.genome.jp/).

### Molecular docking

The protein structures and MENK 2D structures were downloaded separately from the Protein Data Bank (PDB) and PubChem. The MENK 2D structures were then converted to MOL2 format using OpenBabelGUI. Then, we conducted molecular docking and calculated the length of hydrogen bonds. We obtained the ligand and pictures using AutoDock and PyMOL software.

### qPCR analysis

The qPCR system started with reverse transcription reaction at 42 °C for 5 min and 95 °C for 10 s, followed by 40 cycles of PCR reaction at 95 °C for 5 s and 60 °C for 30 s*5, and finally the dissociation protocol was carried out. Primer sequences were in Table 1. The relative amount of gene expressions were calculated using the 2^−△△CT^ method.

**Table 1:** PCR primer sequences

Primer	Direction	Sequence
HO-1	Forward	5’-TCACTTCGTCAGAGGCCTGCTA-3’
	Reverse	5’-AGCGGTGTCTGGGATGAGCTA-3’
FTH	Forward	5’-GTGCGCCAGAACTACCACCA-3’
	Reverse	5’-GAGCCACATCATCTCGGTCAA-3’
GAPDH	Forward	5’- AAATGGTGAAGGTCGGTGTGAAC-3’
	Reverse	5’- CAACAATCTCCACTTTGCCACTG-3’

### Western blot

Equal amounts of protein were separated by SDS-PAGE and then incubated overnight with specific antibodies against HMOX1(1:2000)(Abcam, ab189491)/FTH(1:1500) (Abcam, ab75973)/GAPDH(1:20000)(Affinity, AF7021). After washing, the blots were incubated with a secondary antibody(1:10000) (Immunoway, RS0002). The data were analyzed using ImageJ software.

### Immunofluorescence

The cells were fixed with 4% formaldehyde, permeabilized with 0.5% Triton X-100 and blocked with 10% goat serum (Beyotime, C0265) for 1 h at room temperature, incubated with HMOX1(1:250)(Abcam, ab189491)/FTH(1:100) (Abcam, ab75973) antibody at 4 °C overnight, then added FITC IgG (H + L)(1:1000)(Immunoway, RS0004) at room temperature for 1 h. Finally, we added DAPI to stain the cell nucleus. Images were acquired with a fluorescence microscope (Olympus, Japan) using DP Manager software.

## Result

### Database search

**Fig. 1:**
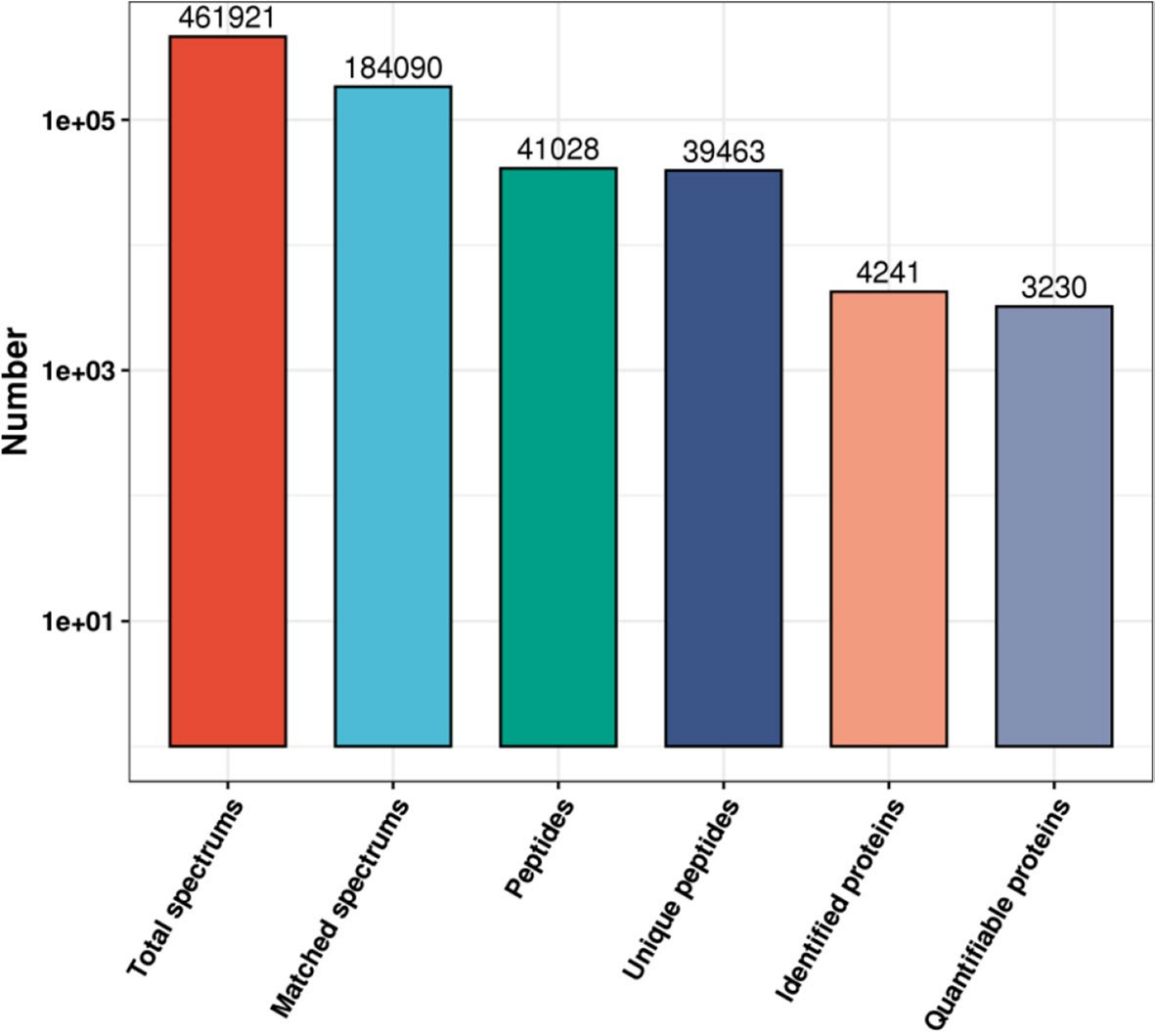
Overview of the mass spectrometry results. Bar plot summarizing the detected peptides and proteins in the normal group and the MENK group

In total, 461,921 spectrograms were obtained using MS. The number of available spectrograms were 184,090, and 39.9% of them were effectively utilized. A total of 41,028 peptides were identified through spectrogram analysis, of which 39,463 were specific peptide segments. A total of 4,241 proteins were identified, of which 3,230 were quantifiable (Fig. 1). The MS/MS result data file was shown in Supplementary Table 1.

### Quality control of data

**Fig. 2:**
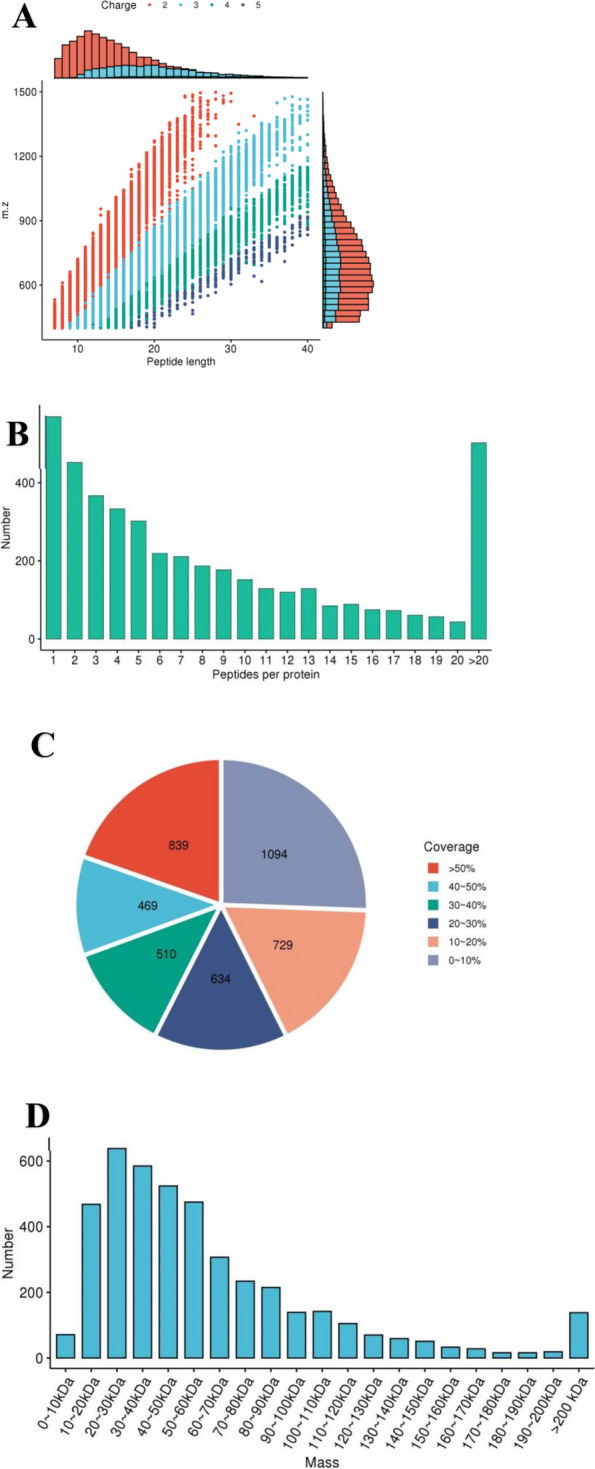
Peptide distribution. **A** Peptide length distribution. Most peptides were distributed in the range of 7–20 amino acids. **B** Peptide number distribution. **C** Protein coverage distribution. **D** Protein molecular weight distribution

After conducting the database search, a series of quality control evaluations were needed to ensure that the results met the required standards. These evaluations included the distribution of peptide length, peptide number, protein coverage, and protein molecular weight (Fig. 2).

### DEP analyses

**Fig. 3:**
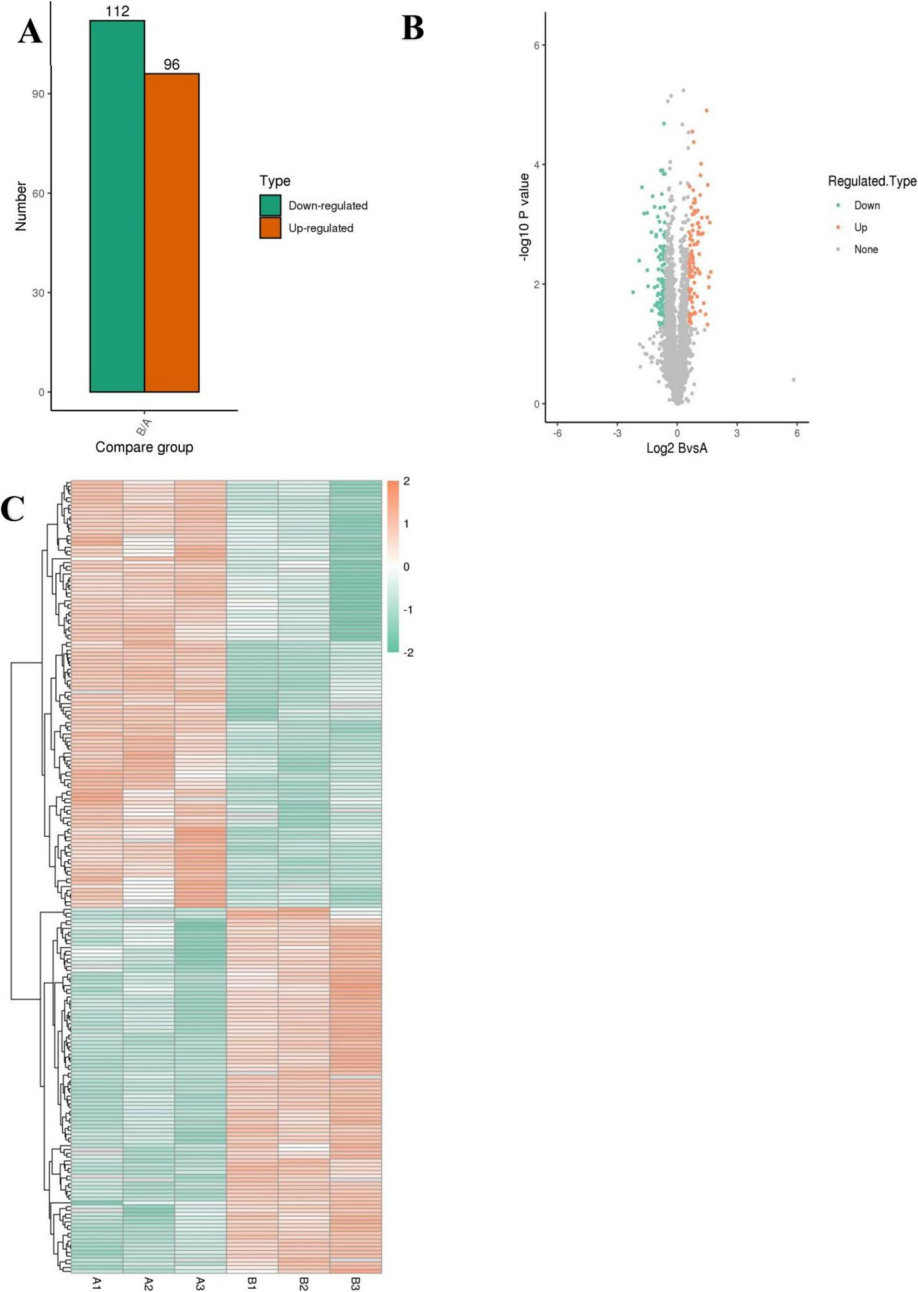
Identification of DEPs between the control group and the MENK group. **A** The total number of upregulated and downregulated DEPs. The significant thresholds for the upregulated differentially expressed proteins (DEPs) were set at a fold change (FC) > 1.50 and a *p*-value (*P*) < 0.05. FC < 0.67 and *P* < 0.05 were set as the significant thresholds for the downregulated DEPs. **B** Volcano plot of the identified DEPs between the control group and the MENK group. The red dots represent upregulated DEPs, the green dots represent downregulated DEPs, and the gray dots represent unchanged proteins. **C** Differential protein heatmap

Compared with the control group, 208 DEPs were identified in the MENK group (Fold change > 1.5): 96 proteins had upregulated expression and 112 proteins had downregulated expression (Fig. 3). The differentially expressed protein summary in Table 2.

**Table 2:** Differentially expressed protein summary

Regulated type	Fold change > 1.2	Fold change > 1.3	Fold change > 1.5	Fold change > 2
Up-regulated	255	178	96	29
Down-regulated	381	278	112	17

### Enrichment analyses of DEPs

**Fig. 4:**
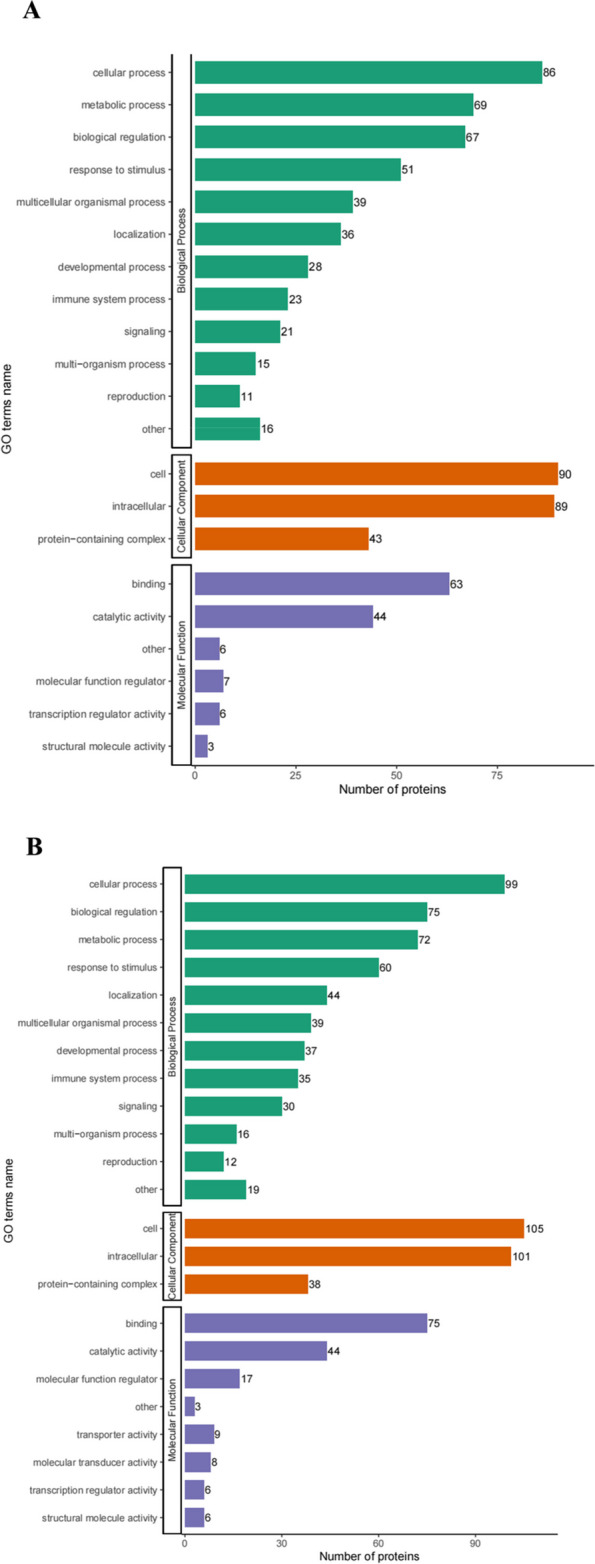
GO analysis for the identified DEPs. The DEPs were annotated into three categories based on GO terms, including biological processes, cellular components, and molecular functions. **A** GO enrichment analysis of the upregulated DEPs. **B** GO enrichment analysis of the downregulated DEPs

**Fig. 5:**
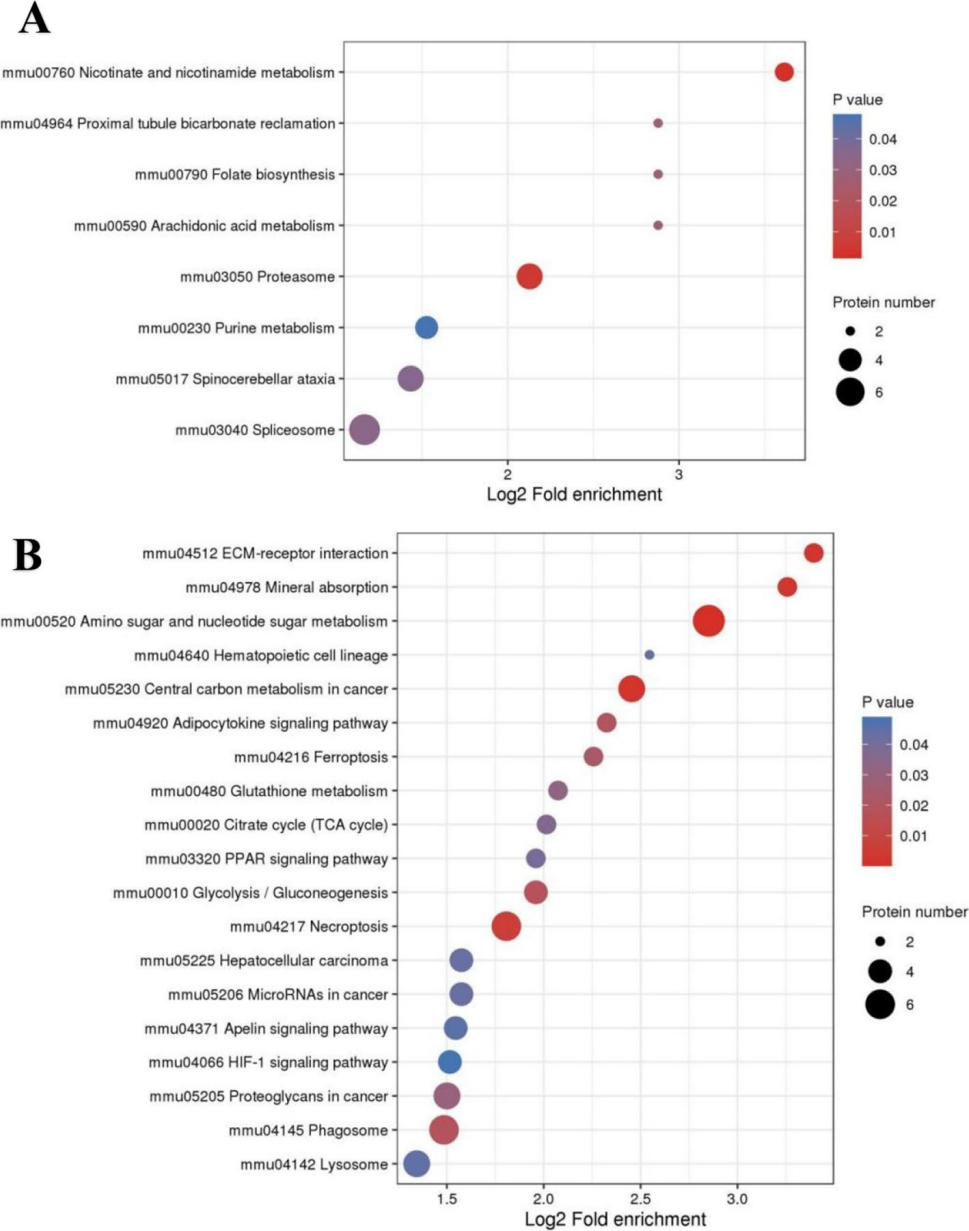
KEGG pathway enrichment analysis of DEPs. Bubble diagrams displaying KEGG pathways for significantly enriched upregulated (**A**) and downregulated (**B**) DEPs. The size of the bubbles describes the number of DEPs in the pathway

GO and KEGG databases were used to ascertain whether DEPs had significant enrichment in specific functional categories. DEPs functional enrichment analysis was conducted in Fig. 4. The enriched KEGG pathways were shown in Fig. 5. Upregulated proteins were detected with proteasome (mmu03050) and nicotinate and nicotinamide metabolism (mmu00760) (Fig. 5A). Downregulated proteins were associated with ferroptosis (mmu04216), necroptosis (mmu04217), mineral absorption (mmu04978), and central carbon metabolism in cancer (mmu05230) (Fig. 5B).

### Hierarchical clustering analysis

**Fig. 6:**
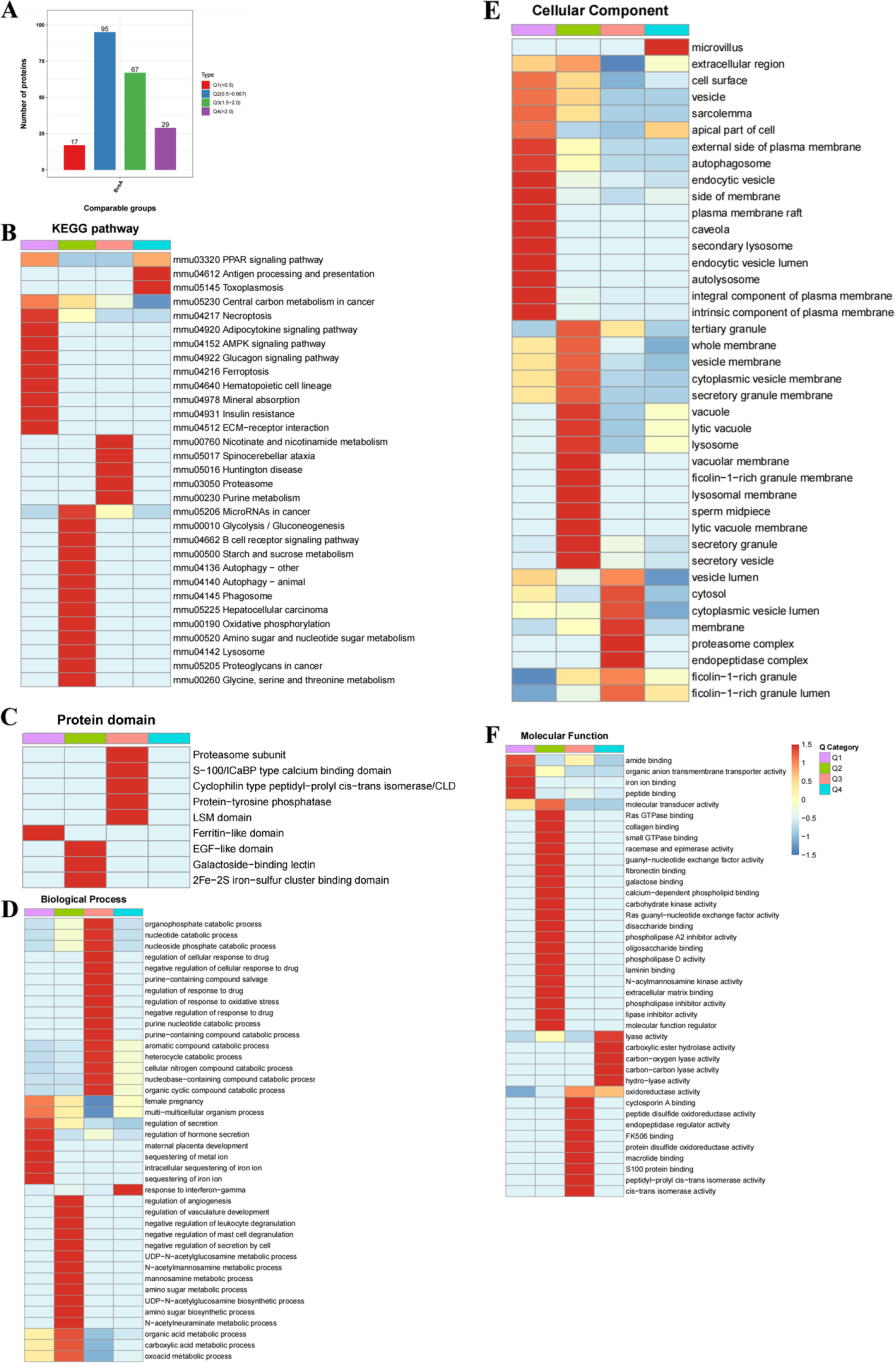
Hierarchical cluster analysis for the DEPs. **A** The significant thresholds for the DEPs were a fold change > 1.50 or < 0.67 and *P* < 0.05. The DEPs were divided into four groups, Q1 to Q4, based on the degree of FC. Q1 (FC ≤ 0.5, severely downregulated), Q2 (0.5 < FC ≤ 0.667, mildly downregulated), Q3 (1.5 < FC ≤ 2.0, mildly upregulated), and Q4 (FC > 2.0, severely upregulated). **B** The Q categories for KEGG pathways. **C** The Q categories for protein domains. **D** The Q categories for biological processes. **E** The Q categories for cellular components. **F** The Q categories for molecular function. The red color indicated a stronger enrichment. The blue color indicated weaker enrichment

According to the degree of fold change, DEPs were categorized into four groups: “severely downregulated” (Q1), “mildly downregulated” (Q2), “mildly upregulated” (Q3), and “severely upregulated” (Q4) (Fig. 6A).

DEPs with significantly downregulated expression were enriched for the pathways related to “ferroptosis”, “necroptosis”, “mineral absorption”, and “central carbon metabolism in cancer” (Fig. 6B, Supplementary Table 2). In the clustering analysis of the protein domain, some DEPs with significantly downregulated expression were enriched for “Ferritin-like domain” and “2Fe-2S iron-sulfur cluster binding domain” (Fig. 6C, Supplementary Table 3). In the clustering analysis using the GO database, DEPs with significantly upregulated expression were enriched for biological processes such as “regulation of response to oxidative stress” and “nucleobase-containing compound catabolic process”. On the other hand, downregulated expression included processes like “regulation of secretion”, “sequestering of metal ion”, and “intracellular sequestering of iron ion” (Fig. 6D, Supplementary Table 4). The main cellular components were enriched for “lysosome” and “ficolin-1-rich granule lumen” (Fig. 6E). The main molecular functions were enriched for “iron ion binding”, “peptide binding”, and “oxidoreductase activity” (Fig. 6F).

### KEGG pathway in iron

**Fig. 7:**
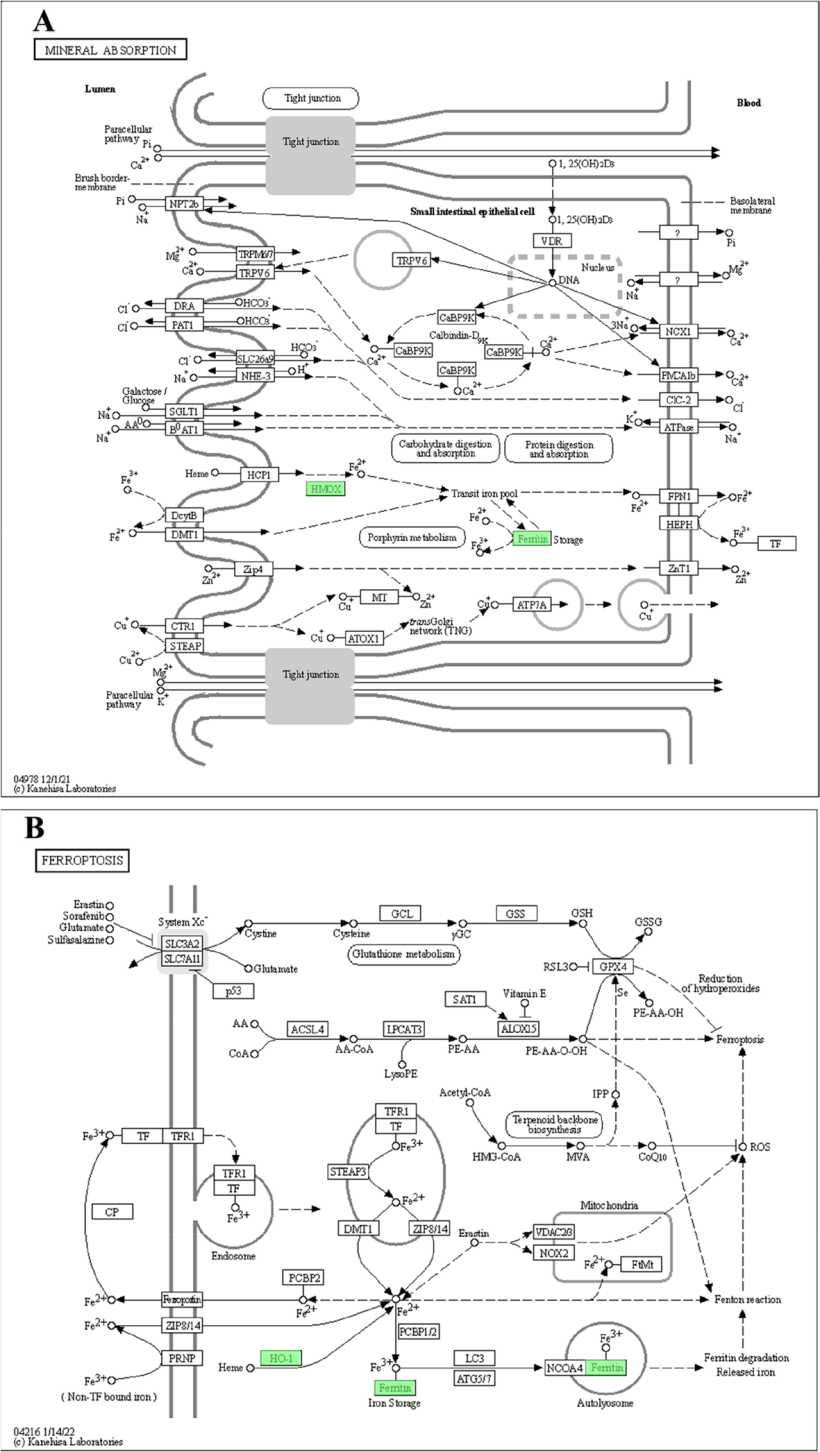
KEGG enrichment pathway. **A** Pathway of mineral absorption. **B** Pathway of ferroptosis. Downregulated proteins were highlighted in green

The KEGG pathway obtained from the enrichment analysis above was visualized as a web page. We have identified two main pathways in ferroptosis and iron metabolism that contain down-regulated genes, HMOX1 and Ferritin (FTH and FTL) (Fig. 7).

### Analyses of protein-protein-interaction networks

**Fig. 8:**
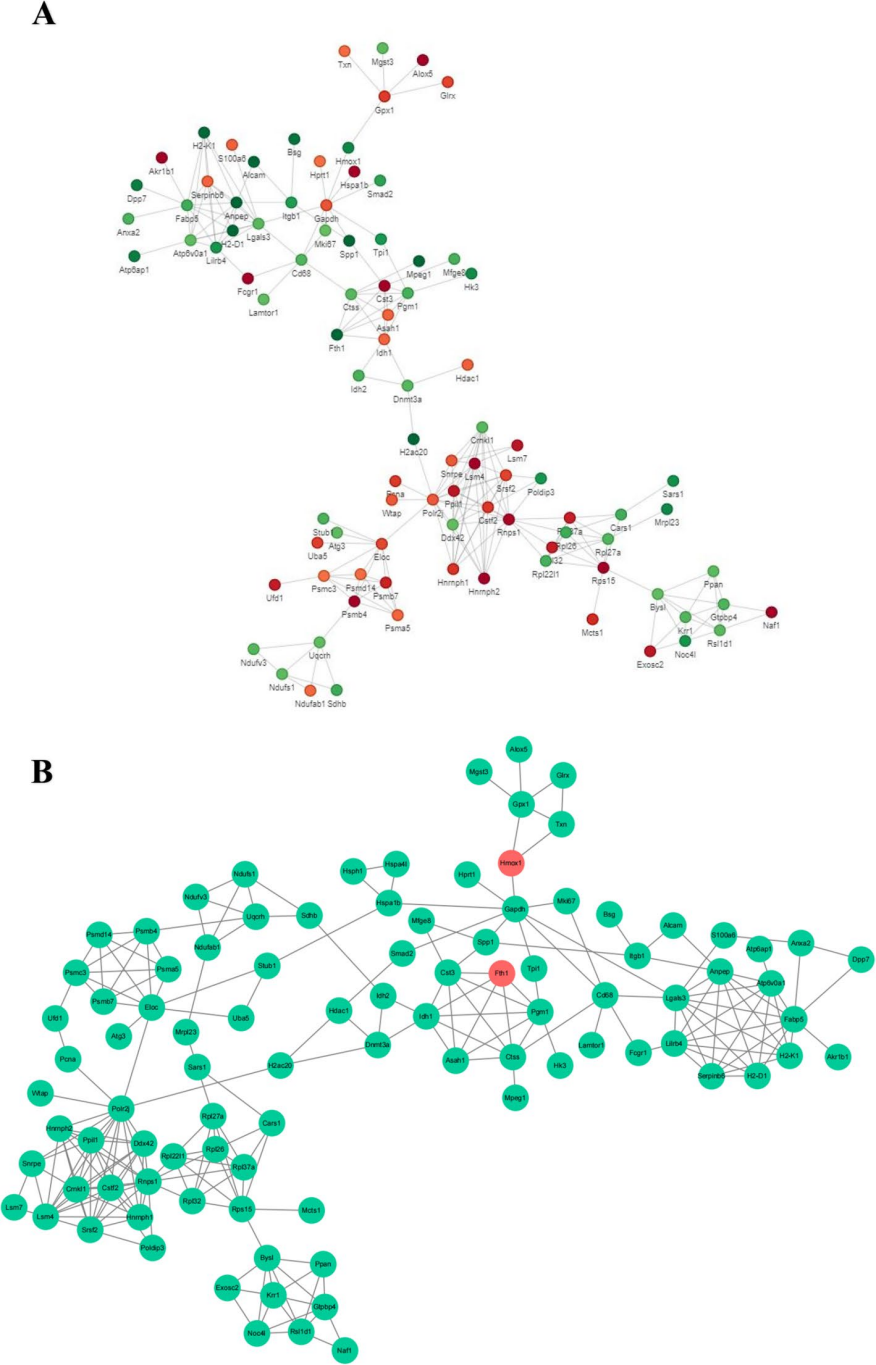
PPI network. **A** Differential expression of proteins. Circles represented differentially expressed proteins, with different colors indicating the direction of protein expression (green for downregulated proteins, red for upregulated proteins). The darker the color, the greater the difference in fold size. **B** Correlation between HMOX, FTH, and their surrounding proteins

In order to clearly demonstrate the interaction relationships between proteins, we screened the top 50 proteins with the closest interactions and constructed the protein interaction network (Fig. 8A). HMOX1 and Ferritin (FTH) were screened in the PPI network shown in Fig. 8B. HMOX1 was found to be connected with GAPDH (upregulated), while FTH was found to be related to Cst3 (upregulated) and Pgm1 (downregulated).

### HMOX1 and Ferritin GO screening

To determine the function of MENK in influencing macrophages through HMOX1 and Ferritin, we conducted a GO enrichment analysis by docking HMOX1 and Ferritin (FTH and FTL). The results were listed in Supplementary Table 5 (HMOX1), Supplementary Table 6 (FTH), Supplementary Table 7 (FTL), and the common GO list (Supplementary Table 8). HMOX1 and Ferritin were enriched in the immune system process and iron ion homeostasis in BP enrichment. HMOX1 was found to be enriched in membrane rafts, caveolae, membrane regions, membrane microdomains, and membranes in the cellular component (CC). We explored how it works in the cytoplasm and the surrounding membrane. MF included phospholipase D activity, phospholipase activity, phosphoric diester hydrolase activity, and lipase activity. FTH and FTL were found to be enriched, suggesting that they may act in the lysosome and extracellular region.

### Molecular docking

**Fig. 9:**
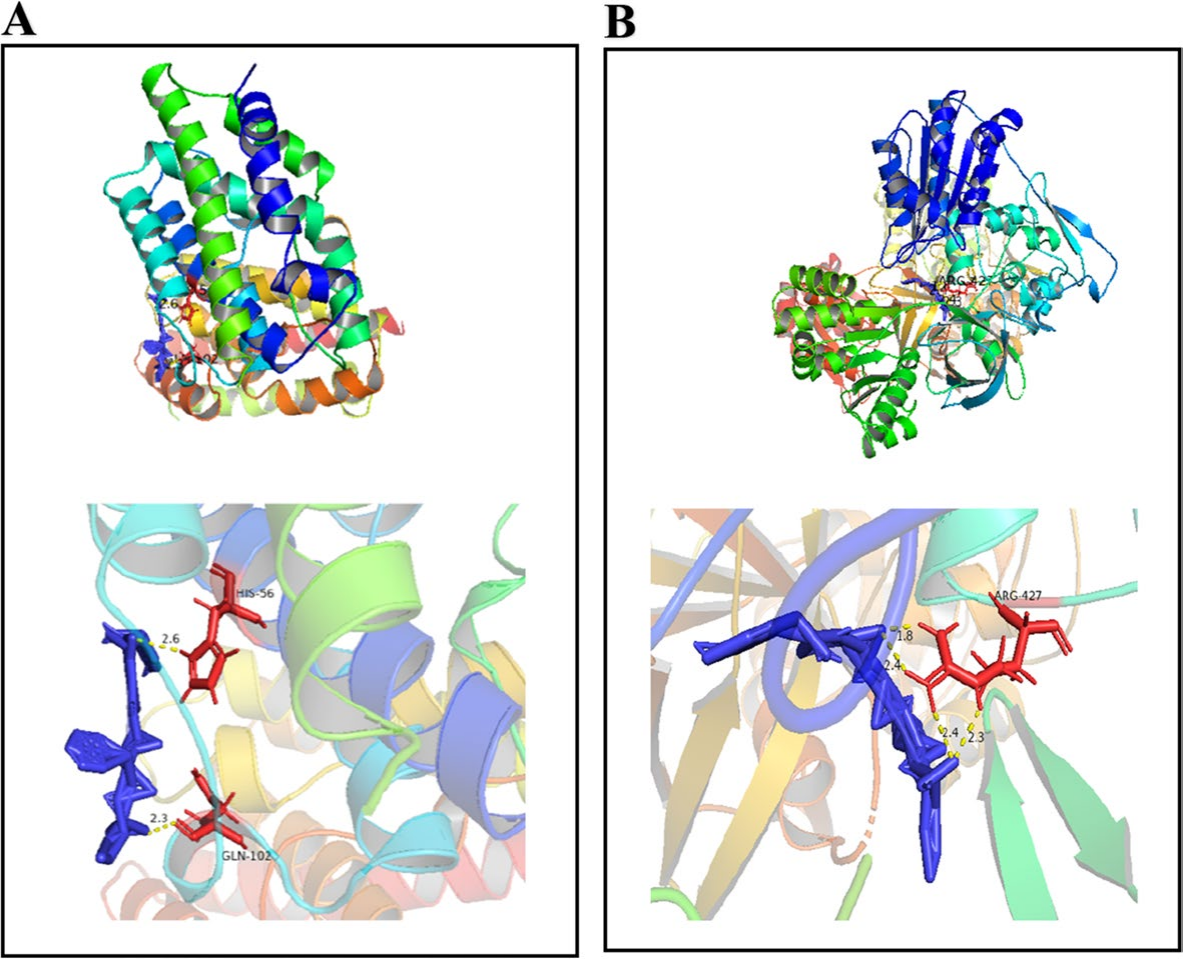
Molecular docking of MENK. **A** MENK-HMOX1. **B** MENK-NADPH

Based on the hub genes in iron-related proteins, crystal structures of HMOX1, NADPH, FTH, and FTL proteins were downloaded from the PDB database. These structures were then improved using PyMOL. Subsequently, molecular docking of the target proteins to the related ingredients was performed using AutoDock. Finally, the PyMOL software was used to calculate the length of hydrogen bonds, enhance the images, and export them. Except for FTL and FTH, HMOX1 and NADPH successfully docked with MENK (Fig. 9). The affinity score was used to calculate the binding ability. And it is generally believed that, affinity <-7 kcal/mol indicates stronger binding activity, -7 kcal/mol suggests moderate binding activity, and affinit > -4 kcal/mol indicates weak binding activity [31]. After calculation, it was found that HMOX1 (-7.0) exhibited stronger binding activity with MENK, while NADPH (-5.78) showed moderate binding activity with it. Finally, the associations of HMOX1 and NADPH with MENK were shown using PyMOL software. The length of hydrogen bonds was calculated. We found two hydrogen bonds between HMOX1 and MENK, and four hydrogen bonds between NADPH and MENK. The hydrogen bonds were represented as yellow dotted lines in the diagrams. In HMOX1, MENK combined with histidine (position 56 in the amino acid sequence) and glutamine (position 102 in the amino acid sequence). In NADPH, MENK combined with the arginine (position at 427 in the amino acid sequence). These results showed that MENK had binding activity with HMOX1 and NADPH, which may be the targets of MENK’s action.

### MENK downregulated HMOX1 and FTH

**Fig. 10:**
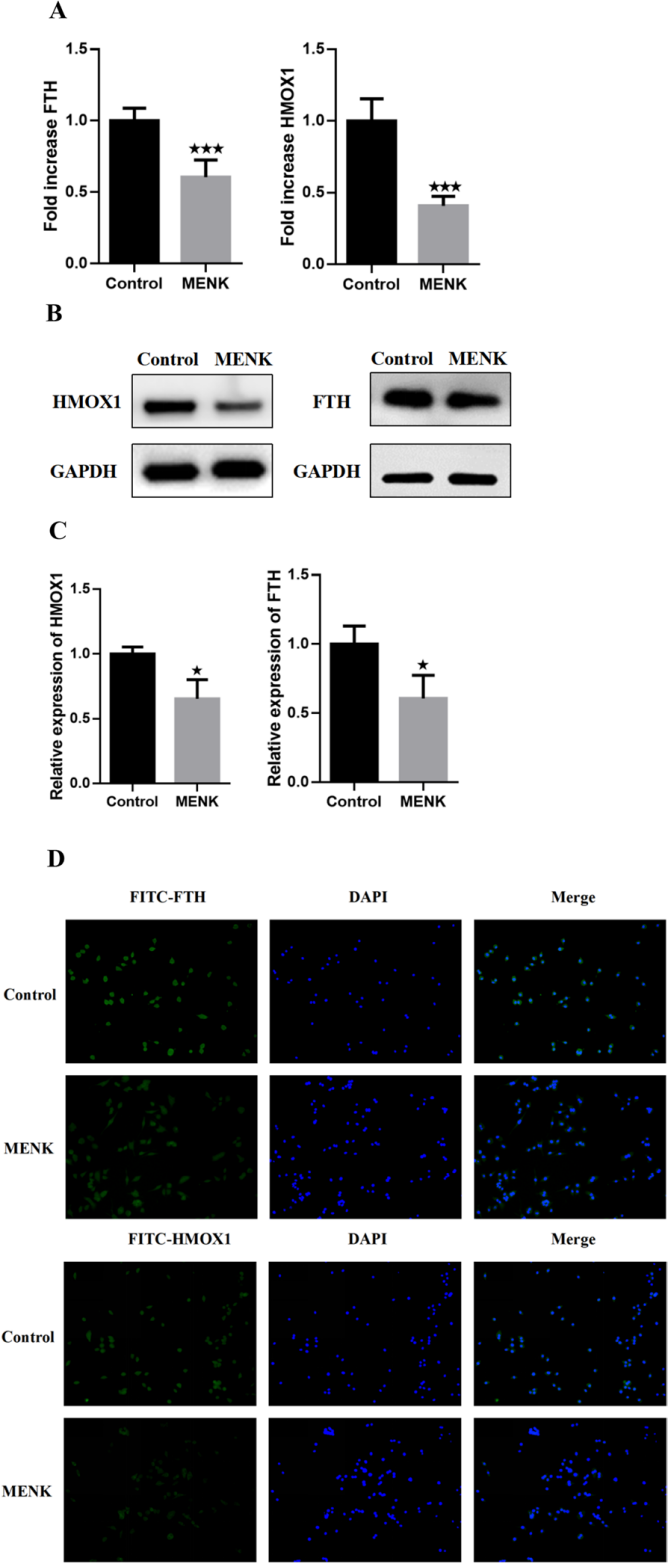
MENK downregulated HMOX1 and FTH. **A** The gene levels of HMOX1 and FTH were quantified at 72 h by qPCR in RAW264.7 cells. **B** and **C** The protein expressions of HMOX1 and FTH were detected at 72 h by Western blot in RAW264.7 cells. The results were presented as fold increase over the control group. **D** The localization and expression of HMOX1 and FTH protein in RAW264.7 cells at 72 h. Data represent the mean ± SD of three independent experiments. ^★^*P* < 0.05, ^★★★^*P* < 0.001 versus the control group

The results of proteomics analysis showed that MENK could downregulated the factors related to ferroptosis, HMOX1 and FTH. Therefore, we measured the levels of HMOX1 and FTH in RAW264.7 cells at 72 h after MENK intervention in macrophages. As shown in Fig. 10A-C, the mRNA levels and protein expression of HMOX1 and FTH decreased to varying degrees after MENK intervention in cells (*P* < 0.001, *P* < 0.05). Further, localization of HMOX1 and FTH by immunofluorescence showed that both intracellular HMOX1 and FTH expressions decreased after MENK intervention (Fig. 10D).

## Discussion

Ferroptosis is a newly discovered concept in 2012, referring to a type of cell death that is dependent on iron. Since the time-transgressive discovery was reported, tumor progression and therapy methods have been propelled into a new era. Various studies have determined that ferroptosis plays a key role in killing tumor cells and inhibiting tumor growth [32, 33]. The study explored whether chemotherapy drugs targeting the progression of ferroptosis have become an important aspect of tumor pharmacology development. According to the results of our proteome and bioinformatics analysis, we have found an intriguing phenomenon. MENK might be a potential regulator intervening in iron ion metabolism. This discovery will further prove the anticancer function of MENK and demonstrate its power in immunotherapy.

In KEGG, we found two key pieces of information: mineral absorption (mmu04978) and ferroptosis (mmu04216). Both of them indicated that iron was involved in the regulation of MENK in the immune microenvironment. Besides, they commonly showed that MENK impacted the association between ferroptosis and macrophages. Both the mineral absorption (mmu04978) pathway and the ferroptosis (mmu04216) pathway showed that HMOX1 was downregulated. The same sign could be found in PPI. Hence, we deduced that HMOX1 seemed to show a potential relationship between MENK and macrophages through iron metabolism and ferroptosis.

Heme-oxygenase (HMOX1, HO) is a cytoprotective enzyme that plays a role in the inflammatory response and the metabolism of heme into pro-oxidant ferrous iron [34]. Some researchers have reported that the anti-inflammatory function of M2 macrophages was revealed through the upregulation of HMOX1 [35, 36]. In the process of ferroptosis, HMOX1 was a positive regulator, particularly in certain cancer diseases [37]. Withaferin A, a natural ferroptotic agent, induced ferroptosis in neuroblastoma by promoting iron accumulation and ROS production through the upregulation of HMOX1 [38]. Many studies have reported that high expression of HMOX1 induces ferroptosis in colon cancer cells, breast cancer cells, and others [39, 40]. Overexpression of HMOX1 was closely associated with ferroptosis.

According to GO enrichment, we found that the biological processes of cellular metal ion homeostasis and iron ion homeostasis were associated with HMOX1. This suggests that MENK regulates iron metabolism to maintain homeostasis. HMOX1 enrichment was observed in various cellular compartments, including membrane rafts, caveolae, membrane regions, membrane microdomains, and the plasma membrane. They were enriched in cytoplasm, surrounding the membrane. MF included phospholipase D activity, phospholipase activity, phosphoric diester hydrolase activity, and lipase activity. These findings demonstrated that HMOX1 influenced lipid metabolism at the membrane, contributing to iron homeostasis. The imbalance between iron-dependent L-ROS accumulation and lipid hydroperoxide (LOOH) detoxification could be a trigger for ferroptosis. This could also explain the connection between lipid metabolism and iron metabolism homeostasis [41].

Binding with PPI, we found that macrophages RAW264.7 interfered with MENK downregulated HMOX1 and upregulated NADPH simultaneously, which interacted with HMOX1. However, a report showed that cisplatin increased NADPH and decreased HMOX1, while TNF-α increased and IL-10 decreased. The use of vanillin in cisplatin resulted in the inhibition of NADPH and the stimulation of HMOX1, which in turn inhibited inflammation [42]. This finding demonstrated that downregulation of HMOX1 stimulated inflammation. Besides, NADPH helped release iron accumulation and ROS production by combining with GSH, which reduced the risk of ferroptosis [43]. In our previous study, the data showed that MENK stimulated RAW264.7 cells’ proinflammatory secretion (IL-6, TNF-α) [30]. It can provide powerful evidence to improve our predictions. In molecular docking, we successfully docked MENK with HMOX1 and NADPH. According to the information mentioned above, we predicted that MENK had a similar function to cisplatin in stimulating inflammation by downregulating HMOX1 expression. Moreover, we deduced that MENK protected macrophages from ferroptosis by downregulating HMOX1. And this might be a possible reason why MENK reduced the death of macrophages, as shown in our previous study.

Besides the downregulation of HMOX1, ferritin also appears to be another factor in regulating iron metabolism in macrophages. Ferritin is a metalloprotein complex that is used to store iron in the cytoplasm [44]. It is made up of 24 subunits, and it has two main subunits: the heavy subunit (FTH) and the light subunit (FTL) [45]. In our GO enrichment analysis, Ferritin (FTH and FTL) was found to be involved in both cellular metal ion homeostasis and iron ion homeostasis biological processes. For Ferritin (FTH and FTL), CC enrichment included autolysosomes, secondary lysosomes, autophagosomes, and lysosomes, among others. MF included iron ion binding. The ferritin-like domain was also enriched with ferritin protein. These findings showed that FTH and FTL played a role in regulating iron metabolism within the lysosome, contributing to iron homeostasis. Alkhateeb AA summarized that ferritin could be detected at higher levels in many cancer patients, and higher expression of ferritin was correlated with more aggressive tumor progression and poorer clinical outcomes [46]. For iron storage, FTH and FTL have distinct functions. FTH has a ferroxidase activity that facilitates the oxidation of Fe^2+^ to Fe^3^+. FTL works in iron nucleation and mineralization. Iron is transported to the lumen of ferritin through pores in the shell. It then reaches the center of the catalytic ferroxidase and subsequently deposit inside in the form of ferrihydrite [42]. However, the high expression of ferritin in the tumor microenvironment, especially in tumor-associated macrophages, may not be a favorable outcome for patient prognosis. It has been recognized as having a close relation to tumor progression or therapy resistance [47, 48]. In our exploration of the KEGG database, we found that MENK inhibited ferritin expression. From KEGG pathways, downregulated ferritin could also be observed in the mineral absorption (mmu04978) pathway and the ferroptosis (mmu04216) pathway. In PPI, the protein associated with ferritin, called FTH, was downregulated. The results of PCR, WB and immunofluorescence confirmed that MENK downregulated the level of HMOX1 and FTH. Therefore, we deduced that MENK might be an effective drug for controlling tumor progression, which could be attributed to its ability to downregulate ferritin in macrophages.

As mentioned above, HMOX1 and ferritin were found in the ferroptosis pathway, regulated iron metabolism and maintain iron homeostasis. Our research provided a novel research direction for understanding the role of MENK chemotherapy in the process of ferroptosis. Further, we will have more work on interaction between HMOX1, FTH, and FTL and whether there is a direct relationship among them, continue to explore regulatory proteins both upstream and downstream to demonstrate the relationship between MENK and ferroptosis. Followed, we will confirm whether the process of ferroptosis can be influenced by MENK in order to induce changes in the macrophages within the tumor microenvironment.

## Conclusion

Our proteomics analysis demonstrated that MENK could be a potential chemotherapy drug by regulating iron metabolism in macrophages. The process involved the downregulation of HMOX1 and ferritin. Our findings provide a new research direction, showing the connection between ferroptosis and macrophages, and suggesting that MENK could be a potential chemotherapy drug in cancer therapy.

### Supplementary Information

**Additional file 1: Supplementary Table 1.** The MS/MS result data file.

**Additional file 2: Supplementary Table 2.** KEGG pathway enrichment.

**Additional file 3: Supplementary Table 3.** Protein domain enrichment.

**Additional file 4: Supplementary Table 4.** GO enrichment.

**Additional file 5: Supplementary Table 5.** HMOX GO enrichment analysis.

**Additional file 6: Supplementary Table 6.** FTH GO enrichment analysis.

**Additional file 7: Supplementary Table 7.** FTL GO enrichment analysis.

**Additional file 8: Supplementary Table 8.** Shared GO enrichment analysis among HMOX, FTH and FTL.

## Data Availability

The data for this study can be found in this article/Supplementary material. Please connect with the author for more details consultation.
